# Human skin bacterial microbiota homeostasis: A delicate balance between health and disease

**DOI:** 10.1002/mlf2.12064

**Published:** 2023-06-04

**Authors:** Yibin Zhu, Xi Yu, Gong Cheng

**Affiliations:** ^1^ Tsinghua University‐Peking University Joint Center for Life Sciences, School of Medicine Tsinghua University Beijing China; ^2^ Shenzhen Bay Laboratory Institute of Infectious Diseases Shenzhen China

**Keywords:** homeostasis, human diseases, skin bacterial microbiota, therapeutics

## Abstract

As the largest organ of the body, the skin acts as a barrier to prevent diseases and harbors a variety of beneficial bacteria. Furthermore, the skin bacterial microbiota plays a vital role in health and disease. Disruption of the barrier or an imbalance between symbionts and pathogens can lead to skin disorders or even systemic diseases. In this review, we first provide an overview of research on skin bacterial microbiota and human health, including the composition of skin bacteria in a healthy state, as well as skin bacterial microbiota educating the immune system and preventing the invasion of pathogens. We then discuss the diseases that result from skin microbial dysbiosis, including atopic dermatitis, common acne, chronic wounds, psoriasis, viral transmission, cutaneous lupus, cutaneous lymphoma, and hidradenitis suppurativa. Finally, we highlight the progress that utilizes skin microorganisms for disease therapeutics, such as bacteriotherapy and skin microbiome transplantation. A deeper knowledge of the interaction between human health and disease and the homeostasis of the skin bacterial microbiota will lead to new insights and strategies for exploiting skin bacteria as a novel therapeutic target.

## INTRODUCTION

**Figure 1:**
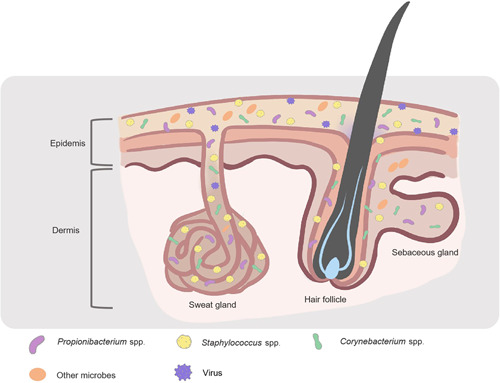
Skin physiology. In human anatomy, the skin comprises two distinct layers: the epidermis and the dermis. The surface of the skin is characterized by its acidity, abundance of salts, dryness, and aerobic conditions. In contrast, the invaginations that make up the sebaceous glandular units of the hair follicles are comparatively anaerobic and even richer in lipids. The makeup of the skin's microbiome is influenced by the chemical environment of the skin's ecotone.

The microbiome plays a critical role in human health and disease^1^, ^2^. The human body is a complex and diverse site of microbial colonization, and these microorganisms help humans maintain physiological homeostasis, prevent pathogen invasion, and educate the immune system to maintain health and homeostasis^3^, ^4^. The skin, being the body's biggest organ, acts as a barrier against diseases and is home to a variety of beneficial bacteria. Skin disorders or even systemic diseases may occur from the breakdown of the barrier or an imbalance between symbionts and pathogens. In human anatomy, the skin consists of two distinct layers: the epidermis and the dermis^5^. The outermost layer of the skin consists of a lipid‐filled and granular‐cornified layer dotted with hair follicles and glands that secrete lipids, antimicrobial peptides (AMPs), enzymes, salts, and many other chemical compounds^6^, ^7^. The skin surface is an acidic, salt‐rich, dry, aerobic environment, whereas the invaginations that form the sebaceous glandular units of the hair follicles are relatively anaerobic and even more lipid‐rich (Figure 1)^8^. The skin, on the other hand, is filled with a variety of different lipids not found elsewhere in the body^9^. For example, triglycerides can be metabolized by microorganisms into free fatty acids, diglycerides, and monoglycerides, which can be biologically active against other microbes or stimulate host cells^10^, ^11^. Sorbic acid is one of these lipids with antibacterial activities^12^.

Human skin areas can be classified according to their physiological characteristics, for example, oily, moist, or dry. On a smaller scale, the skin region's diversity and density of glands and hair follicles differ tremendously, creating a complex physical and chemical landscape known as geographically unique niches. The microbiome's composition is determined by the chemistry of the skin ecotone; nevertheless, there are important variations in composition at the species and strain levels due to unidentified microbial and host characteristics. For certain species, *Acinetobacter* is known to frequently colonize various body parts within the same person^13^, while *Staphylococcus* epidermidis exhibits variations across different body parts among individuals (e.g., differing in the axillae of different individuals)^14^. Additionally, species such as *Corynebacterium, Staphylococcus*, and β‐*Proteobacteria* typically demonstrate a preference for moist areas, such as axillae, elbows, and knee creases^15^. It is vital to investigate the diversity and composition of the microbiota in various skin sites to understand the mechanisms of the pathogenesis of common skin disorders that favor particular skin sites, such as eczema on the inner side of the elbow and psoriasis on the outer side of the elbow^16^, ^17^.

The development of the skin's microbiota commences at birth and lasts for a few weeks, largely depending on the body site^18^. Adolescence leads to significant shifts in the microbiota, with an increase in the dominance of *Corynebacterium* and *Propionibacterium* and a decrease in the quantity of *Firmicutes* (including *Staphylococcus* and *Streptococcus*)^19^. Even though adult skin is constantly exposed to the environment, the microbial composition is relatively stable with time^20^. Therefore, it can be inferred that symbiotic microorganisms and their hosts develop stable, mutually beneficial associations. On the other hand, when the skin is subjected to inflammation, the skin microbiome composition is significantly altered^16^. It is currently unknown how homeostasis is reshaped, how infections interact with the already present symbiotic community, or how pathogens and skin inflammation form a destructive cycle.

Historically, culture‐based methods have been used to define skin microbial communities for functional investigations on the impacts of skin microbiome on human health. Because this approach selects microorganisms that can be cultured under artificial growth conditions, such studies generally fail to see the nonculturable skin microbes and ignore the function of the overall skin microbiome. Therefore, researchers have started to use sequencing techniques, such as 16S rRNA sequencing, to avoid the bias introduced by culturing methods and to accurately reflect the variety of the skin microbiome. With the development of sequencing technologies, the shotgun metagenomics has been widely used to study the human skin microbiome. The majority of human microbiome shotgun metagenomic cataloguing has concentrated on species composition and diversity^21^, ^22^. Recent research, however, has demonstrated that even within a species, the consequences of various strains on the host varied dramatically^23^. However, research on the skin microbiome is still in its early stages, and strain‐level variations are still unknown.

In this review, we outlined the composition of the skin bacterial microbiota and its homeostatic balance in connection to human health. On the other hand, we focused on the interaction between human diseases and the skin bacterial microbiota. A fuller understanding of the relationship between human health and disease and the homeostasis of the skin bacterial microbiota will generate insights and innovative concepts for using skin bacteria as a novel therapeutic target.

## SKIN BACTERIAL MICROBIOTA IN RELATION TO HEALTH

**Figure 2:**
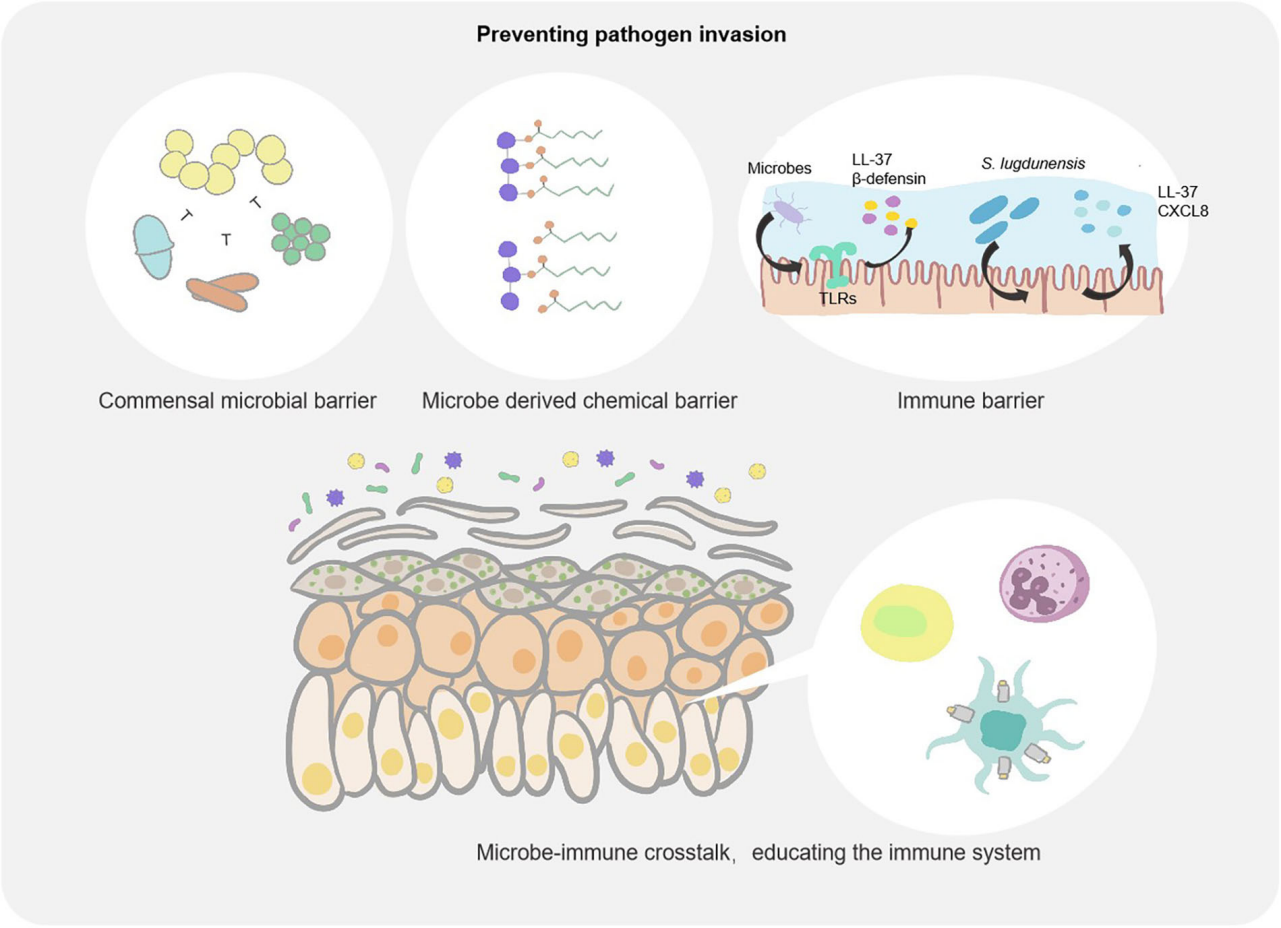
Skin bacterial microbiota in human health. Skin microbial homeostasis is critical in maintaining human health, especially by educating the immune system and preventing pathogen invasion. The cutaneous microbiome and the bioactive molecules produced by microbes constantly educate the immune system. In addition, commensal skin microbes create the first line of defence against pathogen invasion by forming a microbial barrier, boosting the chemical barrier, and stimulating innate and adaptive immune defenses.

Skin microbial homeostasis is critical to the maintenance of human health (Figure 2). Before investigating the effects of skin microbes on human disease, it is necessary to understand the basic composition of the skin bacterial microbiota and its homeostasis in relation to human health. A comprehensive review of the general microbial composition and distribution throughout the skin habitat, compartments, and microenvironment is required since few studies have investigated the skin microbiome as a whole‐body system^24^. Additionally, research on the microbiota's similarities and differences in health disparities, as well as the skin's microbiota's response to various biological events, may provide important implications for disease risk and outcomes^25^.

### The composition of skin bacteria in the healthy state

The skin is the most exposed organ in the body, with an estimated surface area of 1.8 m^2^ (and possibly even more when hair follicle structures and other appendages are taken into consideration). The skin is home to over 1 million bacteria/cm^2^, which collaborate to preserve a physical barrier against the external environment and prevent pathogen penetration and invasion^8^, ^23^, ^26^, ^27^. The skin serves both as a protective barrier and as a vital thermostat for the body. However, the skin should not be thought of as a barrier protecting us from the bacterial communities that inhabit our bodies. The skin bacterial microbiota should be viewed as a comprehensive and dynamic interface where microbes interact with the immune system to carry out vitally important regulatory activities^2^, ^28^, ^29^.

Typically, four phyla, *Actinobacteria* (*Corynebacterium, Propionibacterium, Propionibacterium, Micrococcus, Actinobacterium, Brevibacterium*), *Firmicutes* (*Staphylococcus, Streptococcus, Finegoldia*), *Proteobacteria* (*Paracoccus, Haematobacter*), and *Bacteroidetes* (*Prevotella, Porphyromonas, Chryseobacterium*), represent the majority of skin symbiont bacteria in humans. In addition, up to 4% of the diversity of skin microbes is archaea^30^, ^31^. One of the most numerous phyla with members that may be involved in ammonia oxidation activities is phylum *Thaumarchaeota* ^31^. Cutaneous β and γ human papillomaviruses are frequently found in many people, and other resident and transient viruses are also present in healthy human skin^32^, ^33^. Phages are one of the least‐studied communities on the skin due to their low biomass. Nevertheless, they have the strength to dramatically influence the physiological activity and complexity of the skin's microbiota^34^.

Three physiological variants of human skin can be broadly characterized: (a) oily/sebaceous areas, such as the forehead, upper back, and nose; (b) dry areas, like the forearms and the lower back; and (c) moist areas, like the armpits, backs of knees, nostrils, and groin^5^. A sequencing investigation on healthy adults showed that the physiology of the skin site affects the composition of the skin bacterial microbiota^15^, ^24^. These various skin microenvironments are home to particular bacterial taxa: Sebaceous gland ecotones are clearly preferred by *Propionibacterium*, whereas *Staphylococcus* and *Corynebacterium* species prefer to colonize damp environments, and *Aspergillus* and *Flavobacterium* survive in dry environments^15^. In addition, even in the same areas, skin bacterial microbiota can be strongly influenced by lipid and water levels. For instance, a case study of individual variations in the microbiome of the facial skin revealed that sebum content on the cheeks, but not on the forehead, was substantially linked to microbial composition and diversity. Conversely, the moisture content in the forehead was found to be a reliable indicator of the type and variety of the local microbiota^35^.

Alterations in the skin microbiome are influenced by several factors, including aging, host habits, and the human immune system. Aging is a key factor leading to significant changes in the composition of the skin bacterial microbiota. Multiple studies have observed an increase in species diversity in the cutaneous microbiota of aged individuals^36^, ^37^. Notably, data from the skin bacterial microbiota can predict age more precisely than data from the gut or mouth microbiome^38^. Skin aging is a process that results in structural and physiological alterations in the skin. These alterations are linked to decreased levels of moisture, the emergence of hyperpigmented patches and wrinkles, and changed sebaceous gland activity^39^. The nutrition of commensal bacteria may be reduced, which may promote the colonization of opportunistic species, particularly if sebum production is decreased. In several skin areas, older age groups showed a decline in the predominant genus of dermal bacteria and a simultaneous increase in the relative abundance of *Corynebacterium* and some *Proteobacteria* ^36^, ^37^. In addition, the use of cosmetics may stimulate the diversity of bacteria on the skin, particularly in genera such as *Ralstonia* spp., which has been provisionally related to the capacity to metabolize xenobiotics^40^, ^41^. Another significant characteristic regulating the alterations in the skin bacterial microbiota is the cutaneous immune system. For instance, by assessing skin bacteria samples from people with uncommon monogenic primary immunodeficiency (PID), hyper immunoglobulin E (IgE) syndrome, and Wiskott–Aldrich syndrome, it was observed that, despite different underlying mutations, all diseases were characterized by an eczema‐like skin disorder with reduced T and B cells, increased eosinophilia, and elevated IgE levels^42^. Despite the fact that the types of bacteria inhabiting healthy people's skin were typically similar, PID patients' skin was more environmentally vulnerable and showed less temporal stability^42^. In a separate study, patients with PID caused by mutations in STAT1 or STAT3 exhibited increased colonization of Gram‐negative bacteria on their skin compared to healthy controls. Specifically, *Acinetobacter* spp. were found to be more prevalent, while there was a decreased presence of *Corynebacterium* spp.^43^.

### Skin bacteria educate immune system

From an evolutionary perspective, there is a close coevolutionary relationship between the symbiotic skin microbiome and the immune system. The reciprocal relationship between them maintains the homeostasis of the body surface microbiome and prevents the invasion of pathogens. Some AMPs are predominantly expressed as the first line of defense against pathogens^6^, but the expression of other AMPs can be temporary and is regulated by skin bacterial microbiota members^44^, ^45^. The skin microbiome, epithelial cells, and the immune system carry out physiological functions that benefit the body through effective communication. Keratin‐forming cells can initiate this dialog by sensing microorganisms through pattern recognition receptors (PRRs), particularly pathogen‐associated molecular patterns (PAMPs)^23^. A number of microorganisms, including fungi, bacteria, and parasites, are killed immediately and rendered inactive when AMPs are secreted in response to the binding of PAMPs to PRRs, which triggers an innate immune response.

The immune system is constantly being taught by the cutaneous microbiome (Figure 2)^46^. For instance, the immature immune system permits the colonization of microorganisms without inducing an inflammatory reaction during the postpartum period^47^. The establishment of tolerance in this context relies on regulatory T cells, a specific subset of lymphocytes that migrate into neonatal skin during the post‐natal hair follicle morphogenesis phase^48^. During this first phase of tolerance, various bacteria have been proven to have varying effects on the immune system. For instance, it has been demonstrated that *S. epidermidis* cutaneous colonization increases levels of the cytokine interleukin (IL)‐1α^45^, ^46^, which increases the production of cytokines by skin‐homing T lymphocytes that support host defense and skin inflammation^45^, ^46^. Notably, under steady‐state conditions, the response of effector T cells (Teff cells) to cutaneous microbes occurs in the absence of inflammation. This process is termed “homeostatic immunity”^3^, ^49^. In other words, the immune response to infections takes place within the framework of a larger memory reaction to other microbial antigens. This hypothesis was proven when mice that had been exposed to the bacterium *S. epidermidis* were more resistant to fungus‐ and parasite‐related skin illnesses^45^, ^46^. Conversely, an expected inflammatory response was shown adjacent to interferon‐γ‐producing Teff cells when *S. epidermidis* was administered intradermally (rather than locally) to mice. This response was characterized by infiltrating monocytes and neutrophils^46^. *S. epidermidis* must be counteracted by the immune system in the setting of barrier disruption to persist on the skin's surface as a commensal^50^. The distinct influence of microorganisms on the immune system has been shown in previous studies. A robust system of pathogen control results from the ongoing interaction between the cutaneous immune system and the cutaneous microbiome^27^, ^49^. The microbial components that mediate these behaviors and how the immune system detects their presence require more detailed exploration in upcoming investigations. Additionally, immunological methods for monitoring these symbiosis‐specific immune responses should be developed^51^. These technologies must enable the viewing of microorganisms and immune cells within tissues to ascertain how infection or inflammation affects their location and function. For the transformation from observation to treatment, additional research combining the study of skin barrier function with immunologic and microbiological triggers is essential.

### Skin bacteria prevent pathogen invasion

The ability of skin microorganisms to alter the skin barrier and prevent pathogen entry is another crucial function. The skin is a strong structure comprised of a stratified and cornified epithelium consisting of keratinocytes, which undergo terminal differentiation. Chemical and immunological characteristics that strengthen the skin's capacity to act as a barrier further support these physical structures. The skin bacterial microbiota directly influences the commensal and pathogenic microorganisms that are present on the skin surface as well as all other components of the skin barrier. In this section, we will discuss how skin microorganisms build barriers to prevent pathogens from invading their hosts (Figure 2).

Skin bacteria use a number of colonization‐resistance mechanisms, such as resource exclusion, direct inhibition, and/or interference, to create an initial barrier to the environment. In polymicrobial communities, skin bacterial microbiota competes for resources and have developed strategies to directly combat their rivals. For example, coagulase‐negative *staphylococci* (CoNS) species‐producing antibiotics have substantial inhibitory effects against the predominant skin pathogen. *Staphylococcus aureus*, the main skin pathogen, is inhibited by a variety of CoNS species^52^. In addition, other organisms, such as *Staphylococcus capitis*, compete with *S. aureus* by hindering the quorum‐sensing pathways of the accessory gene regulator (agr), which are necessary for *S. aureus* pathogenicity^53^, ^54^. Notably, several of these antagonistic processes cooperate with the host's defense against microbes. For instance, the *Staphylococcus lugdunensis* peptide antibiotic lugdunin causes keratinocytes to secrete the AMP LL‐37 and the neutrophil chelator CXCL8 through the TLR‐MyD88 (Toll‐like receptor‐myeloid differentiation main response protein) pathway (Figure 2)^55^. CoNS species in the skin bacterial microbiota are not the only ones affected by the competitive process. The human wrinkle unit is a competitive environment for *Propionibacterium* acnes, and some strains produce the thiopeptide antibiotic cutimycin to reduce *S. aureus* colonization^56^. Uncertainty persists over how these individual interactions come together in the context of the community and how this influences the structure and operation of the community. The skin physical barrier and epithelial differentiation are both influenced by the skin microbiome. The stratum corneum is constantly in direct contact with the microbiota (resident or temporary) and is also regulated by microbes. Sphingomyelinase, which transforms lamellar lipids into ceramides^57^, a crucial element of the stratum corneum, is secreted by cutaneous microorganisms. Furthermore, by creating lipases that degrade sebaceous triglycerides into free fatty acids, microbes also contribute to the chemical barrier of the skin by keeping it more acidic and preventing the colonization of invasive and pathogenic species. The acidic skin surface not only inhibits bacterial colonization physically through keratinocytes and skin lipids but also chemically through the creation of a hostile environment. Both *Cutibacterium acnes* and *Corynebacterium* species secrete lipases that degrade triglycerides in sebum to produce free fatty acids^58^, ^59^. Free fatty acids directly inhibit bacteria and promote the expression of human beta defensin 2 (hBD‐2) to further improve skin immunity^60^.

Additionally, bacteria trigger the production of AMPs and proteins, which serve as natural antibiotics, as well as innate and adaptive immune responses. When TLR signaling is activated by microbial signals, the production of the protein fragment AMP LL‐37, which is a fragment of the cathelicidin protein, increases (Figure 2)^61^. The skin also generates AMPs from the β‐defensin family, which are bactericidal against *S. aureus* and *Escherichia coli*, in addition to the cathelicidin family AMPs. The small proline‐rich proteins SPRR1 and SPRR2 are secreted by the sebaceous glands in response to Gram‐negative bacterial lipopolysaccharides, and they directly disrupt negatively charged bacterial membranes^62^. Additionally, the skin generates a large number of cationic intrinsically disordered proteins with antimicrobial effects^63^. These AMPs act synergistically to enable a variety of antimicrobial defenses against environmental microbes in the skin.

## SKIN BACTERIAL MICROBIOTA IN RELATION TO DISEASE

**Figure 3:**
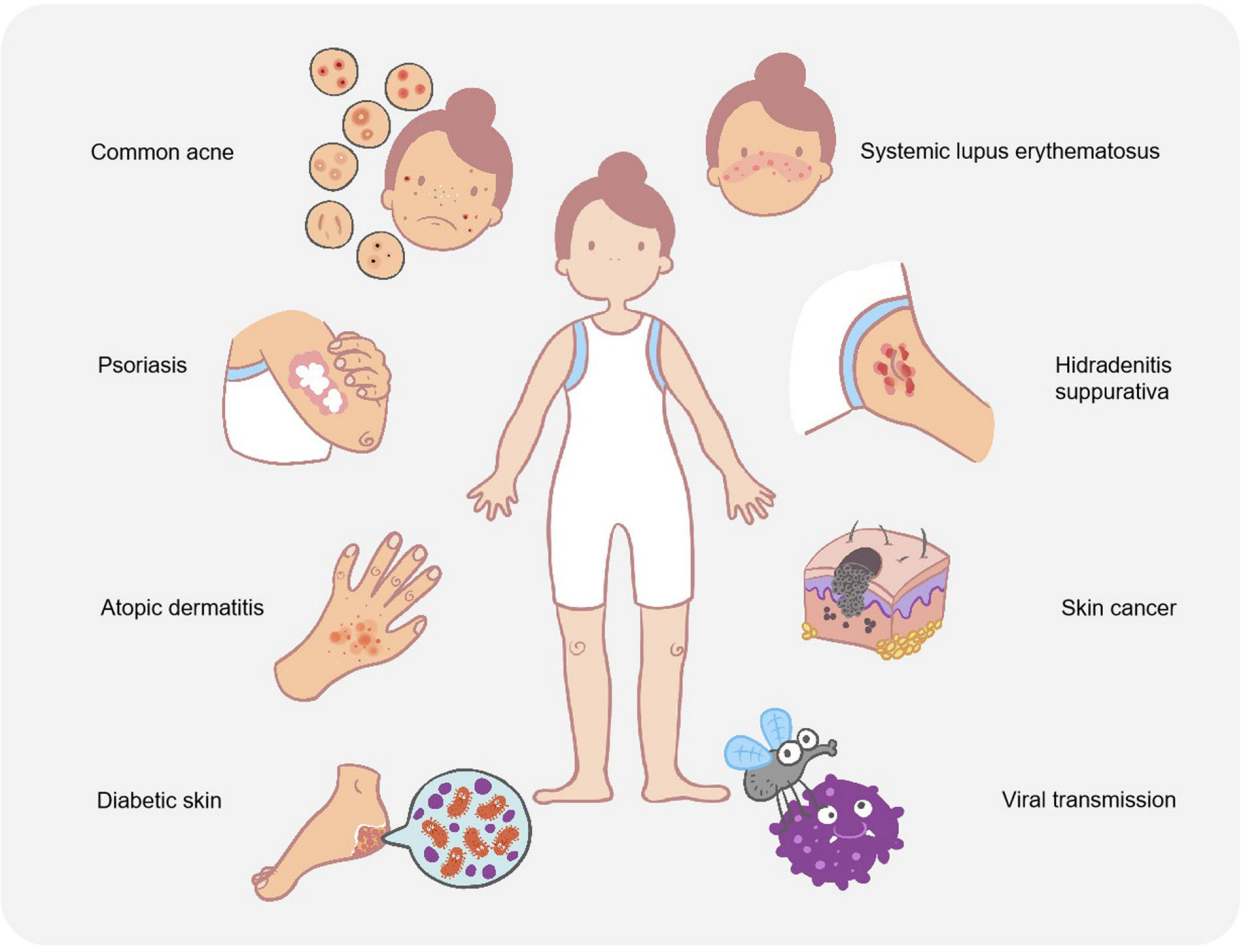
Skin bacterial microbiota in human disease. Skin microbial disorders in humans can lead to various diseases, including atopic dermatitis, common acne, chronic wound infections, psoriasis, viral transmission, systemic lupus erythematosus, hidradenitis suppurativa, and skin cancer.

Interactions between microorganisms on the body surface shape the symbiotic microbiome of human skin and prevent the colonization of pathogenic bacteria. However, in some specific cases, disturbances of this symbiotic microbiome may lead to the development of human diseases. Many common skin diseases, such as acne, eczema, chronic wound healing, and other skin diseases, are associated with changes in the microbiome and are called microbiome disorders also known as “dysbiosis”. This disorder is driven by common commensal microorganisms (Figure 3). Recently, altered microbiome homeostasis of surface microorganisms caused by viral infections has also been found to result in increased host attraction to mosquitoes, thereby facilitating virus transmission. Furthermore, clinical observations have revealed that in addition to common bacterial infections, many viruses can cause severe skin diseases and even develop into cancer. In this section, we will discuss how dysbiosis is responsible for certain diseases, as well as its relationship with viral infection and transmission. Understanding the mechanisms underlying skin bacterial microbiota‐influenced diseases and the interactions between skin microbiome and viral infections will potentially enable new strategies for disease treatment and virus control.

### Skin bacterial microbiota dysbiosis and atopic dermatitis (AD)

Dry skin, itchy patches, and reoccurring eczema are the hallmarks of AD, a chronic skin disease that can affect multiple body regions^64^. AD has a prevalence of 15%–20% in children and a lower prevalence (3%) in adults^65^, ^66^. Low bacterial diversity is often associated with AD, and skin bacterial microbiota dysbiosis may play a pivotal role in the pathogenesis of eczema. Bacterial populations were found to be more diverse and abundant in preclinical and posttreatment skin than they were in eczema‐prone skin areas^4^, ^16^.

**Table 1:** The key microbial findings in skin diseases.

Diseases	Key microbial findings
Atopic dermatitis	Increased *Staphylococcus aureus* levels are associated with atopic dermatitis
	*Staphylococcus epidermidis, Cutibacterium acnes*, and *Corynebacterium* spp. usually act against *S. aureus* invasion
	Decreased *Lactobacillus* species and *Finegoldia* species promote potential infections
Common acne	*C. acnes* and *Malassezia* are associated with the emergence of acne vulgaris
Chronic wound	*Pseudomonas aeruginosa* and anaerobic bacteria are generally detected with deep chronic ulcers
	*S. aureus* is typically associated with acute superficial ulcers
Psoriasis	Decreased microbial diversity
	Higher abundance of *Corynebacterium* in psoriasis
	Lower abundance of *Propionibacterium* in psoriasis
Viral infection	*S. aureus* is shown to increase herpes simplex virus (HSV) infection in keratinocytes
	Dengue and Zika virus disrupt host skin microbes to promote viral transmission

Reduced antimicrobial protein production has been correlated with changes in the commensal microbiota^4^, ^67^, which is typically triggered by CoNS, such as *S. epidermidis* ^68^, ^69^. Antimicrobial activity is typically present in CoNS strains isolated from healthy individuals, while it is infrequently present in AD patients. Increased *S. aureus* colonization correlates with decreased numbers of antimicrobial‐producing CoNS, and reintroduction of these strains into AD skin decreases the abundance of *S. aureus* ^52^. Skin flares from AD are commonly linked with more *S. aureus* abundance^70^, whereas the abundance of *S. epidermidis* ^71^, *C. acnes* ^64^, and *Corynebacterium* spp.^72^, which usually acts against *S. aureus* invasion^73^, decreases (Table 1). *S. aureus* was more prevalent in the dermis of lesions, indicating that deeper skin layers were more easily accessible during flares^74^. In addition, *S. aureus* occurred in 70% of lesioned skin and 39% of non‐lesioned or healthy skin^69^. Non‐lesioned skin has a similar structure to healthy skin and, therefore, a more similar microbiota^16^. Therefore, numerous disease features have been indicated by the oscillatory detection of *S. aureus* in AD lesions^71^. AMPs are usually produced by human cells; however, the expression of particular AMPs is decreased in AD patients, indicating an absence of the ability to protect against pathogens such as *S. aureus* ^52^. The pathogenesis of AD is greatly aided by the presence of *S. aureus* in lesions and the consequent absence of commensal‐produced AMPs due to the decreased quantity of commensal bacteria^73^. It is reasonable to hypothesize that commensal communities promote skin health by producing antimicrobial activity, the absence of which exacerbates the disease^52^. In addition, skin barrier deficiencies, related to AD, can compromise the integrity of the lamellar membrane, altering the skin's microbiomes and potentially allowing harmful bacteria like *S. aureus* to thrive. Specifically, *S. aureus* produces Sal2/Geh lipase and active proteases, which can further disrupt the lipid and corneocyte barriers and compromise the skin's defenses^75^. Furthermore, it appears that AD involves more than just *Staphylococcus* spp. According to a recent study, the absence of strictly anaerobic bacteria such as *Lactobacillus* and *Finegoldia* species causes the AD microbiota to switch toward a more aerobic metabolism^71^. These microorganisms play a role in keratinocyte AMP responses, which are attenuated in skin with the filaggrin protein mutation^76^. Therefore, the absence of anaerobic microorganisms may decrease crucial skin barrier activities and promote potential infections^71^. In general, the microenvironments in AD that selectively target microbes are associated with alterations in host gene expression, which in turn are influenced by the microbiota^71^. Skin homeostasis depends on this intricate host–microbe interaction, including interactions between *S. aureus* and AD host cells in particular^73^, as ecological dysregulations contribute to the development of disease^16^.

### Skin bacterial microbiota dysbiosis and common acne

More than 9% of the world's population develops a skin lesion, called acne vulgaris, with a prevalence of 80%–90% in adolescents and young adults^77^, ^78^. Acne, papules, pustules, and cysts can all develop as a result of the obstruction and inflammation of the sebaceous units of the hair follicles that induce this type of skin disease^79^. Studies have revealed that certain strains of *C. acnes* and *Malassezia* are associated with the emergence of acne vulgaris, a disease with a complex pathophysiology^80^. Almost all adults have *C. acnes* in their skin, but only a minority suffer from acne because the gene expression profile of *C. acnes* is different in individuals with and without acne^81^, and the abundance of *C. acnes* can instantly increase in individuals with common acne compared to healthy controls, according to various case–control studies^82^. It is interesting to note that microbiome research has also revealed that acne is related to the virulence of the *C. acnes* strain rather than the total amount of *C. acnes* ^80^, ^83^, ^84^. By collecting a sample of the follicular sebaceous units on the noses of the 49 acne patients, differences in *C. acnes* at the strain and genome levels were studied (compared with 52 healthy individuals). Despite similar relative *C. acnes* abundance, metagenomic analysis revealed that the strain population structure in the two cohorts was markedly different. Some strains were highly related to acne, while others were more abundant in healthy human skin^80^. Studies examining the microbiome proportions after systemic antibiotic therapy, however, discovered a decrease in *C. acnes* after various treatment periods, along with an increase in microbial diversity, which increases the possibility that the pathogenesis of acne may be influenced by *C. acnes* abundance and microbiome diversity. This work highlights the significance of a greater comprehension of microbiota diversity in preserving skin microbiome homeostasis for the purpose of studying human skin diseases.

### Microorganisms in diabetic skin and chronic wound infections

In developed nations, chronic wounds are an increasing public health concern. Treatment for wounds that progress into more severe chronic phases can be challenging. Amputation may be necessary in extreme circumstances such as diabetic foot ulcers. Normally, skin breakdown results in an inflammatory cascade activity; however, this immune response is disrupted in diabetic skin^85^. Additionally, a changed microbiome may exacerbate the severity of the disease. The skin bacterial microbiota of people with diabetes in its early stages is quite similar to that of healthy people. Dynamic alterations with changed species diversity and abundance may occur as the disease worsens^86^. Overall, the skin bacterial microbiota of the diabetic foot is less diverse than that of the healthy foot, even though the presence of the most abundant genera has not changed. The differences between the skin bacterial microbiota of the diabetic foot and healthy foot are caused by a decrease in microbial abundance. The skin of both healthy and diabetic feet contained organisms from the three major genera *Staphylococcus* spp., *Acinetobacter* spp., and *Corynebacterium* spp. The healthy skin also had *Kocuria* spp. and *Micrococcus* spp., while the diabetic foot skin also had *Enterobacter* spp.^87^. The skin microbiome of the diabetic foot, however, is less diverse despite numerous similarities. As a result, changes in the less common microbial species, the majority of which are only found in healthy foot skin, can be used to predict whether someone has diabetes^87^. Compared to healthy feet, diabetic foot skin often has low levels of *Staphylococcus* spp. and has a higher proportion of *S. aureus* ^88^. An unbalanced skin bacterial microbiota caused by the high presence of *S. aureus* may cause inflammatory changes and increase the risk of skin infections^89^. Accordingly, *Pseudomonas aeruginosa* and anaerobic bacteria are generally detected with deep chronic ulcers^90^, ^91^, but *S. aureus* is typically associated with acute superficial ulcers^92^ (Table 1). In other words, the abundance of *Staphylococcus* spp. and anaerobic bacteria had a negative correlation with wound depth. Additionally, there was a positive correlation between wound lengthening, microbial diversity, and *P. aeruginosa* abundance, but a negative correlation with *S. aureus* abundance^93^. The healing of substantial chronic ulcers in the inflammatory phase is hindered by this variance in the microbial composition of the two most prevalent species: *S. aureus* and *P. aeruginosa* ^94^, ^95^. Studies comparing the microbiomes of diabetic patients with and without chronic infections could provide information about diagnostic markers that could be utilized as indicators of the probability of developing chronic wounds^85^. These features can be further used to personalize antibiotic therapy and treatment strategies^89^. Use of probiotics to treat common inflammatory skin disorders such as acne vulgaris, AD, and hidradenitis suppurativa (HS) has been suggested and even done successfully^96^. To prevent diabetic patients from developing chronic foot ulcers, similar topical probiotic treatment approaches may help restore a healthy microbiota^89^.

### The role of the skin microbiome in the pathogenesis of psoriasis

Patients with psoriasis develop modest to persistent skin plaques as a result of their chronic inflammatory skin disease. A complicated combination between numerous hereditary and environmental variables that causes an overactive inflammatory response in the skin is thought to be the etiology of this condition. The potential impact of microbiota on its pathogenesis has been studied in recent years (Table 1).

There is evidence from numerous microbiome studies that psoriasis patients' skin may have probiotic problems. In fact, psoriatic skin samples had significantly less microbial diversity than normal skin samples in terms of alpha diversity and beta diversity^97^. Comparing skin lesion samples to nonlesion and control samples, it is interesting to note that the richness and variety were markedly reduced. Psoriasis samples in particular showed a significant univariate decrease in the relative abundance and high taxonomic performance of the genera *Cupriavidus, Flavisolibacter, Methylobacterium*, and *Schlegelella* in comparison to controls^97^. Overall, numerous studies have suggested that the genus *Corynebacterium* may play a role in psoriasis pathogenesis^71^, ^98^. According to several microbiome investigations, compared to normal skin and controls, psoriatic lesions had larger abundances of *Corynebacterium* and lower abundances of *Propionibacterium*. Additionally, *Corynebacterium* and *Propionibacterium* were related to different aspects of aberrant skin capacitation and the severity of local lesions, respectively. The data also revealed a significant association between the regional severity index score for psoriasis used to evaluate the condition and the presence of *Staphylococcus* spp. and *Corynebacterium. Corynebacterium* was thought to have the potential to interfere with the interferon signaling system, which could result in a dysbiosis of the skin microbiome^99^, ^100^.

### Viral infection influences skin microbiome homeostasis

From a clinical point of view, in addition to bacterial pathogens, there are many viral and fungal skin pathogens. Human papillomaviruses and herpesviruses are two examples of viruses that can cause severe skin diseases but often remain latent throughout life. It has been determined by analysis of pertinent double‐stranded DNA virome studies that papillomaviruses and poxviruses can colonize their hosts asymptomatically^101^. Previous skin infection with *S. aureus* has also been observed clinically as a major risk factor for the development of eczema herpeticum with eczema zoster in patients with AD^102^. Alpha toxin from *S. aureus* has been shown to increase the number of herpes simplex virus (HSV) in keratinocytes^103^. Alpha‐toxin‐enhanced HSV replication is blocked in a proteolytic element and metalloproteinase 10 (ADAM‐10)‐deficient keratinocyte, suggesting that alpha‐toxin‐mediated viral enhancement is a receptor‐dependent event and that alpha‐toxin may be required through ADAM 10‐mediated binding to host cells^104^. There is an urgent need to better understand how these viruses interact with the skin's immune system and microbiome, given that herpesviruses, papillomaviruses, and polyomaviruses that infect epithelial cells are responsible for a large number of chronic skin diseases and, in some cases, aggressive skin cancers.

### Virus hijacks the skin microbiome to promote viral transmission

A class of mosquito‐borne, single‐stranded, positive‐stranded RNA flaviviruses (e.g., dengue virus, Zika virus) also causes cutaneous diseases such as rashes, which, in severe cases, can lead to severe cutaneous hemorrhage and even death from hemorrhage shock^105^. These infections are transmitted primarily by vector mosquitoes, which feed on blood and can transmit pathogens once they feed on a host. Numerous pathogens, including those that can cause lymphatic filariasis, malaria, dengue fever, chikungunya fever, encephalitis, and microcephaly disease, are known to be carried by mosquitoes^106^, ^107^. The transmission of these diseases by mosquitoes is significantly influenced by skin microbes. The skin bacterial microbiota produces odors that make us smell differently and render us more or less attractive to blood‐sucking arthropods^108^, ^109^. Among these arthropods, mosquitoes (*Culicidae*) primarily rely on scents to find their hosts, especially volatile organic compounds, but other sensory cues are thought to be crucial for triggering host‐seeking activity in these insects^108^, ^110^, ^111^. In a recent interesting study, mosquito‐borne flaviviruses were found to emit an odor that attracts mosquitoes and promotes virus transmission by manipulating the microbial composition of the host's skin. Hosts infected with dengue or Zika viruses have been found to be more attractive to mosquitoes. Further mechanistic studies revealed that such viruses disrupt host skin microbes by inhibiting RELM‐Alpha^112^, an important AMP in host skin, leading to increased attractiveness to mosquitoes and enhanced viral transmission. This study demonstrated for the first time how a four‐way “virus–bacteria–insect vector–mammal” interaction network promotes disease transmission^113^ and proposed a novel mosquito‐borne disease control strategy that targets the reshaping of the skin microbiome to reduce host attractiveness to mosquitoes and thus inhibit mosquito‐borne virus transmission (Table 1). In addition, there is still great uncertainty about the impact of virus–skin bacterial microbiota interactions on host skin pathology and disease development. By gaining a thorough understanding of the “skin bacterial microbiota–virus–host” interaction, our knowledge of the influence of surface microbiota on human diseases will be substantially expanded, and it has the potential to aid in disease prevention and save lives.

### The role of the skin microbiome in the other skin diseases

In addition to common skin diseases, including AD, psoriasis, acne, chronic ulcers, and viral infections, skin microbiome dysbiosis is also associated with other conditions, such as systemic lupus erythematosus (SLE), a multifactorial autoimmune disease that frequently presents with cutaneous manifestations in up to 85% of cases. A recent study has revealed various bacterial taxa, including *S. aureus* and *S. epidermidis*, as potential markers for SLE skin lesions^114^. Another immune‐related skin disease, HS, is characterized by nodules and abscesses in intertriginous anatomical locations. Although it is now understood that the disorder is an autoinflammatory condition rather than an infectious disease, bacteria still play a role in disease processes. Metabolomic profiling has revealed that the skin bacterial microbiota commonly metabolizes host tryptophan into indoles by activating the aryl hydrocarbon receptor. This mechanism plays a crucial role in regulating tissue inflammation associated with HS, which frequently results in the development of lesions^115^.

Given the link between skin microbial dysbiosis and chronic inflammation and inflammation‐mediated skin diseases, it is not surprising that skin microbiomes have been linked to the development of certain skin cancers. For example, a recent study found that *S. epidermidis* produces 6‐*N*‐hydroxyaminopurin (6‐HAP), which can inhibit DNA synthesis and show antitumor activity on transformed tumor cells. In a mouse model, colonization with a strain of *S. epidermidis* that can release 6‐HAP reduced the incidence of UV‐induced tumor growth compared to a control strain^116^. Malignant melanoma (MM), which accounts for 75% of all skin cancer‐related deaths, is the most serious type of skin cancer among the various types^117^. In a recent study of 27 skin melanoma patients using culture‐based microbial investigation, *Corynebacterium* was found to be more significantly associated with stage III/IV MM patients than with stage I/II MM patients^118^. Moreover, patients with *Corynebacterium* had more IL−17‐positive cells than those without this species. These results imply that IL‐17 may be a key factor through which *Corynebacterium* species regulate the development of MM^118^. Another study using a mouse model revealed that an intratumoral injection of *C. acnes* drastically reduced tumor size and suppressed the development of melanoma cells by enhancing the Th1 immune response^119^.

## EFFORTS TO MANIPULATE THE SKIN BACTERIAL MICROBIOTA

With intensive research on skin microbes, it has been found that an increasing number of diseases are associated with skin microbiome abnormalities. The manipulation of the human skin bacterial microbiota to cure skin diseases has recently given rise to completely new treatment options. Therapeutic strategies based on manipulating the skin microbiome include skin bacteriotherapy, skin microbiome transplantation, and others. The skin microbiome is a new therapeutic platform and the value of applying a single strain or a full microbiome transplant has already been shown in therapeutic applications.

### Bacteriotherapy

As mentioned earlier, AD is closely related to the skin microbiome. The lack of AMPs in skin cells can lead to an imbalance in protection against *S. aureus*. According to previous studies, a healthy microbiome can selectively protect against *S. aureus* through the release of AMPs^120^, ^121^. Comparing the microbiomes of AD patients with lesions, it was found that those who were colonized with *S. aureus* exhibited a less diverse range of taxa in their microbiome, as opposed to patients without *S. aureus* colonization. The aforementioned problem may be related to an inactive antimicrobial defense mechanism in AD patients. The ability of CoNS (*S. epidermidis* and *Staphylococcus hominis*) to produce AMP prompted researchers to isolate CoNS strains from AD patients that prevented *S. aureus* growth. These strains were then multiplied in preparation for eventual autologous transplantation to the skin of subjects and resulted in lower *S. aureus* colonization^52^. In a double‐blind, randomized clinical study, *S. epidermidis* strains were initially isolated from participants. These isolated strains were then cultured and applied twice weekly to the individual's own facial skin for a duration of 4 weeks. In comparison to the control group, the application of *S. epidermidis* resulted in improvements in the relative lipid and moisture content of the patient's skin, while reducing skin moisture evaporation. In addition, after receiving *S. epidermidis*, patients' skin acidity decreased from pH 5.5 to 5, which may have been caused by the patients' elevated levels of lactic and propionic acids^122^. This study suggests that applying *S. epidermidis* to facial skin may have a beneficial impact.

### Skin microbiome transplantation

Are the alterations in the microbiome the result of past exposure or environmental factors? Researchers moved bacterial communities from the tongue to the forehead or palm side of the forearm or from the palm side of the forearm to the forehead or tongue, and then tested them after 2, 4, and 8 h to search for an answer to this question. The bacteria from the tongue were found to colonize on the forearm when transplanted there, but the forearm's natural microbial composition did not change appreciably as a result. The reverse was also true. This suggests that the bacterial composition of a site is largely dependent on its native state^24^. Several studies have shown that mutualistic symbiosis is important for maintaining metabolism between species. We need to focus not only on the transfer of microbiome but also on potential cross‐feeding and coinhabitation. There have been several studies in which the entire original skin microbiome was transferred from one skin site to another. Researchers transferred bacteria capable of producing odor in the armpit to the subject's forearm to determine whether the foul odor would be replicated. Strong odors were produced by the dual bacterial group samples cultivated on the forearm, indicating that bacteria that cause odors can spread from the armpit to the forearm^123^. This study suggests that reshaping human odor and reducing the attractiveness to vectors of infectious diseases by skin microbiome transplantation, thereby blocking virus transmission, provide a novel avenue for infectious disease prevention and control.

## PERSPECTIVES

The significance of skin microbial homeostasis in both health and disease states is outlined in this review. The physiological processes of the human body are maintained in large part by the skin bacterial microbiota. Currently, a growing number of researchers are focusing on the skin microbiome. Future work will require a deeper understanding of the molecular basis of skin microbial homeostasis in the regulation of human disease development and pathogenesis. This includes interactions that are not limited to microbe–microbe interactions, microbe–host–microbe interactions, environmental factor–microbe interactions, and the impact of variability among different bacterial strains on host health. On the other hand, scientists must develop more precise microbiome detection techniques. Using these approaches, new microbes inhabiting the skin's surface and deeper layers will be identified. The advancement of DNA sequencing makes use of increasingly detailed and extensive knowledge regarding the microbiome of the human skin. The role of the skin bacterial microbiota in promoting disease progression and preserving human health is still unclear in many aspects. The skin is a dynamic and stable ecosystem where interactions between skin microbes are strictly regulated. In fact, the sequencing of skin microorganisms contributes to our understanding of human health and disease. Determining which bacterial metabolites and microorganisms are critical for maintaining human health and disease is a topic that must be addressed promptly. Functional studies of skin microorganisms are currently confined to culturable bacteria. However, the inconsistent growth rates of different bacteria on culture media result in some potential functional bacteria not being effectively isolated for further research. For a more comprehensive functional study of skin microorganisms, the isolation of important skin microorganisms by various culture isolation methods or other methods, such as coculturing, will require additional investigation and consideration in the future.

As we learn more about how symbiotic bacteria train the immune system, by taking advantage of the ability of skin microbes to penetrate the skin barrier, we may be able to construct microorganisms capable of transporting cytokines, small chemicals, or vaccinations to specific populations of activated immune cells. With a more thorough understanding of the intricate network of microbe–microbe interactions, we will also be able to provide more specialized therapeutic approaches to control disorders of the skin bacterial microbiota. Dysbiosis of the skin bacterial microbiota has been investigated in relation to its effects on AD and the spread of mosquito‐borne flaviviruses such as dengue virus and Zika virus, but it is possible that the disruption of the skin bacterial microbiota is also a significant contributing factor to many other skin diseases, such as psoriasis, pyogenic dermatitis, and lupus erythematosus. The use of microorganisms to treat dysbiosis in the gut, such as fecal transplants to treat *Clostridium difficile* infections, has been effective^124^, and the bacterial‐based prevention of neonatal sepsis has been developed using *Lactobacillus plantarum* synbiotics^125^. Similar live skin microbial therapies have not yet been developed. However, there is great potential to exploit the immunomodulatory and antimicrobial properties of cutaneous commensal bacteria^45^, ^126^. Manipulation of skin bacteria to control mosquito‐borne viral transmission also holds good promise. Although our knowledge of the association between the human skin microbiome and mosquito attraction is still developing, it is becoming increasingly clear that altering the microbiome of the human skin may present novel options to prevent the spread of vector‐borne diseases. In addition, because of the specific chemical environment of the skin, small‐scale changes in the amount of some nutrients may significantly affect the composition or functionality of the skin microbiome. If certain changes are regulated rationally, they may enable us to control the skin bacterial microbiota and maintain the homeostatic balance of human skin microbes.

The gut–skin axis is another significant part of microbial–host interactions that has not been studied in this field. Numerous bacteria that live in symbiotic associations with humans can be found in both the skin and the gut. For example, the effects between the microbiota in the gut and the host response on the skin are unclear. AD, psoriasis, acne vulgaris, dandruff, and even skin cancer are all closely correlated with altered immune responses and skin disorders that are caused by dysbiosis of the skin or gut microbiome^127^. Immune checkpoint inhibition has been proven to be effective in treating a number of skin cancers that were once known to have a high death rate, such as metastatic melanoma, squamous cell carcinoma, and Merkel cell carcinoma^126^, ^128^. Interestingly, the effectiveness of distant anticancer immunotherapy has been associated with symbiotic gut microbiome^129^. In contrast, there are still knowledge gaps in skin‐resident microbes that affect systemic or distant immune responses, and this topic merits further study. Increased systemic inflammation, such as atherosclerotic cardiovascular disease, has now been linked to processes that were initially assumed to involve only skin‐limited inflammation, such as plaque psoriasis^130^.

In fact, a wide variety of immune cells enter and exit the skin both at rest and during inflammation^131^, ^132^, ^133^. These findings unveil an additional layer of complexity in the intricate interactions between microbes and their hosts. They highlight the importance of not solely focusing on interactions within the local microenvironment but also considering the migration of immune cell populations influenced by the microbiota, microbial products, and metabolites to other areas of the body during microbial stimulation at various barrier sites. This suggests that the impact of the skin bacterial microbiota on the immune system may have broader systemic implications that merit further investigation in future research.
